# Metabolomics Analysis Identifies Differential Metabolites as Biomarkers for Acute Myocardial Infarction

**DOI:** 10.3390/biom14050532

**Published:** 2024-04-29

**Authors:** Jie Zhou, Hai-Tao Hou, Yu Song, Xiao-Lin Zhou, Huan-Xin Chen, Li-Li Zhang, Hong-Mei Xue, Qin Yang, Guo-Wei He

**Affiliations:** 1 Department of Cardiac Surgery & The Institute of Cardiovascular Diseases, TEDA International Cardiovascular Hospital, Tianjin University, Tianjin 300457, China; zhoujie1999@tju.edu.cn (J.Z.); houht@tedaich.com (H.-T.H.); chenhx@tedaich.com (H.-X.C.); zhangll13389962059@163.com (L.-L.Z.); xuehm@tedaich.com (H.-M.X.); yangq@tedaich.com (Q.Y.); 2 Tianjin Key Laboratory of Molecular Regulation of Cardiovascular Diseases and Translational Medicine, Tianjin 300457, China; songy@tedaich.com (Y.S.); zhouxl@tedaich.com (X.-L.Z.); 3 Department of Cardiac Surgery & The Institute of Cardiovascular Diseases, TEDA International Cardiovascular Hospital, Chinese Academy of Medical Sciences, Tianjin 300457, China; 4 Department of Cardiology & The Institute of Cardiovascular Diseases and the Critical Care Unit, TEDA International Cardiovascular Hospital, Tianjin University, Chinese Academy of Medical Sciences, Tianjin 300457, China

**Keywords:** acute coronary syndrome, STEMI, NSTEMI, biomarker, metabolomics

## Abstract

Myocardial infarction (MI), including ST-segment elevation MI (STEMI) and non-ST-segment elevation MI (NSTEMI), is still a leading cause of death worldwide. Metabolomics technology was used to explore differential metabolites (DMs) as potential biomarkers for early diagnosis of STEMI and NSTEMI. In the study, 2531 metabolites, including 1925 DMs, were discovered. In the selected 27 DMs, 14 were successfully verified in a new cohort, and the AUC values were all above 0.8. There were 10 in STEMI group, namely L-aspartic acid, L-acetylcarnitine, acetylglycine, decanoylcarnitine, hydroxyphenyllactic acid, ferulic acid, itaconic acid, lauroylcarnitine, myristoylcarnitine, and cis-4-hydroxy-D-proline, and 5 in NSTEMI group, namely L-aspartic acid, arachidonic acid, palmitoleic acid, D-aspartic acid, and palmitelaidic acid. These 14 DMs may be developed as biomarkers for the early diagnosis of MI with high sensitivity and specificity. These findings have particularly important clinical significance for NSTEMI patients because these patients have no typical ECG changes.

## 1. Introduction

Acute coronary syndrome (ACS) is characterized by the sudden decrease in blood supply to the heart [1], including myocardial infarction (MI) and unstable angina (UA). According to the data list of ARIC research in NHLBI from 2005 to 2014, the annual incidence of MI was 605,000 new cases and 200,000 recurrent cases [2]. Every year, more than 800,000 people experience acute MI, and 27% of them die (most of them before they arrive at the hospital) [3]. In 2020, the death toll from any reported MI reached 158,724 [4].

MI can be divided into ST-segment elevation myocardial infarction (STEMI) and non-ST-segment elevation myocardial infarction (NSTEMI). Usually, complete occlusion of the coronary artery leads to myocardial tissue damage and an increase in cardiac troponin level, leading to STEMI [5]. NSTEMI occurs when coronary artery is not completely occluded by thrombosis or extensive collateral circulation (or both), leading to a subendocardial infarction. Except that there is no acute ST segment elevation or Q wave, its performance may be the same as STEMI. Furthermore, there may be ST segment depression and/or T wave inversion [3].

Among them, STEMI accounts for about 40% of all MI [6]. However, STEMI is more acute and has more severe symptoms than NSTEMI. In addition, the hospitalization risk of STEMI is higher than that of NSTEMI, including death (6.4% for STEMI and 3.4% for NSTEMI), cardiogenic shock (4.4% and 1.6% respectively), and bleeding (8.5% and 5.5% respectively) [4]. Although the morbidity and mortality of STEMI have decreased with the progress of science and technology and the development of the medical industry, it is still an important factor of morbidity and mortality in the world and the main cause of premature death worldwide [3,4,6].

Metabolomics on the biomarkers of STEMI has been conducted. It was found that serum acylcarnitine is related to the clinical manifestations and severity of coronary artery disease [7]. Another study reported that seven metabolic biomarkers and six clinical indicators may predict the hospitalization outcome of STEMI patients [8]. Furthermore, 9-cis retinoic acid was related to the phenotype of the STEMI main left lesion [9]. And 9-cis retinoic acid and dehydrophytosphingosine levels were reported as new biomarkers of early ventricular fibrillation after STEMI [10]. In addition, lysophosphatides can be used as a predictive marker of STEMI and NSTEMI [11].

On the basis of the above-mentioned, biomarkers that may be used for efficient and accurate early diagnosis of MI would be important in the clinical setting, particularly for NSTEMI that does not have early or typical ECG changes. The present metabolomics study conducted a non-targeted screening of differential metabolites of MI, followed by a validation in a new cohort of patients in order to search new biomarkers that may be used for early diagnosis of both STEMI and NSTEMI.

## 2. Materials and Methods

### 2.1. Study Design and Population

This study was approved by the Institutional Review Board of the TEDA International Cardiovascular Hospital in Tianjin, China, and informed consent was obtained from all the patients included in the study. All procedures followed were in accordance with the ethical standards of the responsible committee for human experimentation (institutional and national) and with the Helsinki Declaration of 1975, as revised in 2000.

From February 2022 to April 2023, plasma samples were prospectively collected from patients diagnosed with STEMI, NSTEMI, and UA. STEMI referred to MI in patients with chest discomfort or other ischemic symptoms, who had developed new ST-segment elevations in two contiguous leads or new bundle branch blocks with ischemia repolarization patterns. Patients who presented without ST-segment elevation were designated as having NSTEMI [12]. Patients without ST-segment elevation and who exhibited symptoms suggestive of cardiac ischemia without elevated biomarker values were diagnosed as having UA [12,13]. The inclusion and exclusion criteria of STEMI, NSTEMI, and UA patients were according to the guidelines outlined by the 2017 European Society of Cardiology (ESC) [14].

The control subjects that demonstrated normal coronary arteries underwent a conventional coronary angiography for atypical chest discomfort [15]. In brief, patients’ plasma samples with a negative coronary angiography and no major diseases were used as controls.

After considering the differences in the five cardiac biomarkers (see Table 1) and the onset time of 2–8 h as the sample selection criteria, then matching the sex, age, and race of the control group and the experimental group, a total of 66 patient samples (48 in the discovery phase and 18 in the verification phase) were finally selected for this research.

**Figure 1:**
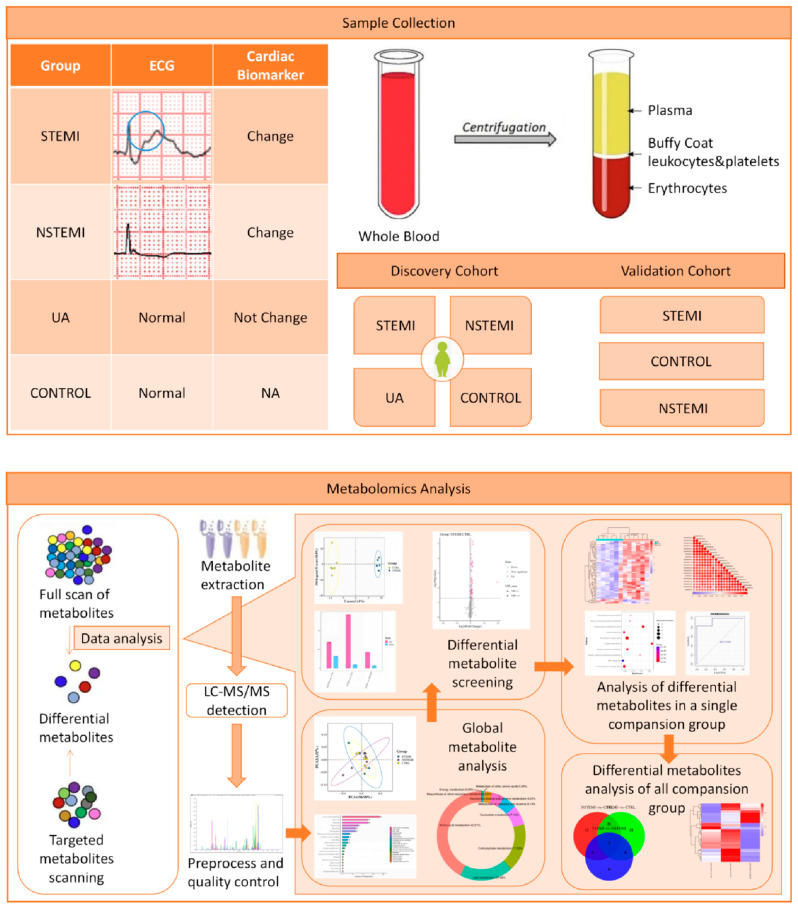
Workflow for the metabolomics study. Firstly, plasma samples were collected, and non-targeted metabolomics was used to identify differential metabolites (DMs) in the STEMI, NSTEMI, UA, and CONTROL groups. After bioinformatics analysis and literature search, some of the DMs were selected and validated by targeted metabolomics in the STEMI, NSTEMI, and CONTROL. The validation results were shown by grouping comparison chart and ROC curve chart in other figures.

The demographic and clinical data of the patients included in this study were taken from the patients’ medical records. The most relevant clinical variables, such as gender, age and race, and possible clinical features, such as body mass index, risk factors, cardiac biomarkers (Myo, CKMB, hs-cTnI, BNP, D-Dimer), and laboratory biochemical test parameters, are shown in the Table 1. The experimental workflow is summarized and shown in Figure 1.

### 2.2. Samples Collection

Blood samples, each with a total volume of 2 mL, were collected by venipuncture from the patients. The collected blood samples were centrifuged (1500× *g*, 10 min, 4 °C). The separated plasma and blood cells were stored in their respective freezing tubes at −80 °C until use.

### 2.3. Metabolomics Analysis

#### 2.3.1. Identification of Differential Metabolites (DMs)

The plasma samples were analyzed by metabolomics. The details of the methods were described in the previous studies [16,17,18,19]. Metabolites were extracted, detected by LC-MS/MS, and then analyzed. The DMs between the two biological groups were screened by variable important in projection (VIP), fold change (FC), and *p* value. In this study, VIP ≥ 1, FC ≥ 1.2 or ≤0.83, and *p* < 0.05 were simultaneously satisfied. Cluster analysis, correlation analysis, enrichment analysis, and ROC analysis, etc., were carried out on the DMs in each comparison. The non-targeted OMICs study had four groups including the STEMI, NSTEMI, UA, and CONTROL (CTRL) groups (*n* = 12 in each group).

#### 2.3.2. Validation of DMs

After the DMs were identified from the metabolomics analysis, selected DMs were then verified by targeted LC-MS/MS. 

#### 2.3.3. Bioinformatics Analysis

The identified metabolites were annotated by classification and function to understand the classification and functional characteristics of DMs using the KEGG and HMDB databases. The functional annotation of pathways was made through the KEGG PATHWAY database to determine the main biochemical metabolic pathways and signal transduction pathways that the DMs were involved in.

### 2.4. Statistical Analysis

For the baseline data of the patients, the classified variables were displayed in numbers and summarized as percentages before being compared across the different groups by the Chi-squared test or Fisher exact test. Continuous variables were expressed as the median (95% CI) and interquartile range (IQR). One-way ANOVA, Welch one-way ANOVA, or the Kruskal–Wallis test were used for overall analysis, and Tukey HSD, Games–Howell, or Dunn’s test were used for multiple hypothesis test when appropriated. 

The correlation analysis of DMs was conducted by calculating the Spearman’s correlation coefficient. Regression analysis was used to determine whether there was a significant correlation between the metabolites and their related factors.

The KEGG pathway enrichment analysis of DMs was carried out using the KEGG database and compared with all identified metabolites. A value of *p* < 0.05 is considered statistically significant.

In this study, all charts were statistically analyzed and visualized by WPS Office (v11.1.0.10228-release, Kingsoft, Beijing, China), IBM SPSS Statistics (v26.0, IBM Corp., Armonk, NY, USA), R software (v4.2.1, R Foundation for Statistical Computing, Vienna, Austria), and the related software packages. 

## 3. Results

### 3.1. Baseline Characteristics of Patients

The baseline characteristics of patients are shown in Table 1 and Supplementary Table S1. There were no significant differences in age, sex, body mass index (BMI), and risk factors (including hypertension, dyslipidemia, and smoking) among the STEMI, NSTEMI, UA, and CTRL groups (*p* > 0.05), while more STEMI, NSTEMI, and UA patients had diabetes (*p* < 0.05). As for the current biomarkers used clinically, there were no significant differences among the STEMI, NSTEMI, and UA groups in BNP and D-Dimer (*p* > 0.05), while there were significant differences in Myo, CKMB, and hs-TnI between STEMI and UA (*p* < 0.001) and between NSTEMI and UA (*p* = 0.006), although there was no significant difference between STEMI and NSTEMI (*p* > 0.05). The levels of plasma glucose, alanine aminotransferase (ALT), aspartate aminotransferase (AST), AST/ALT, total protein (TP), and high-density lipoprotein cholesterol (HDL-C) in the STEMI, NSTEMI, and UA groups were changed in comparison to the CTRL (*p* < 0.05). Furthermore, compared with the CTRL, NSTEMI, and UA groups, the levels of ALT, AST, and AST/ALT in the STEMI group were significantly higher. The levels of total protein (*p* = 0.028) and triglycerides (TG) (*p* = 0.03) were significantly different between the CTRL group and UA group. Furthermore, compared with the CTRL group, the levels of HDL-C in patients in the NSTEMI (*p* = 0.008) and UA (*p* = 0.006) group decreased.

### 3.2. Untargeted Metabolomics Profiling of Plasma from Discovery Phase

#### 3.2.1. Screening of DMs

**Figure 2:**
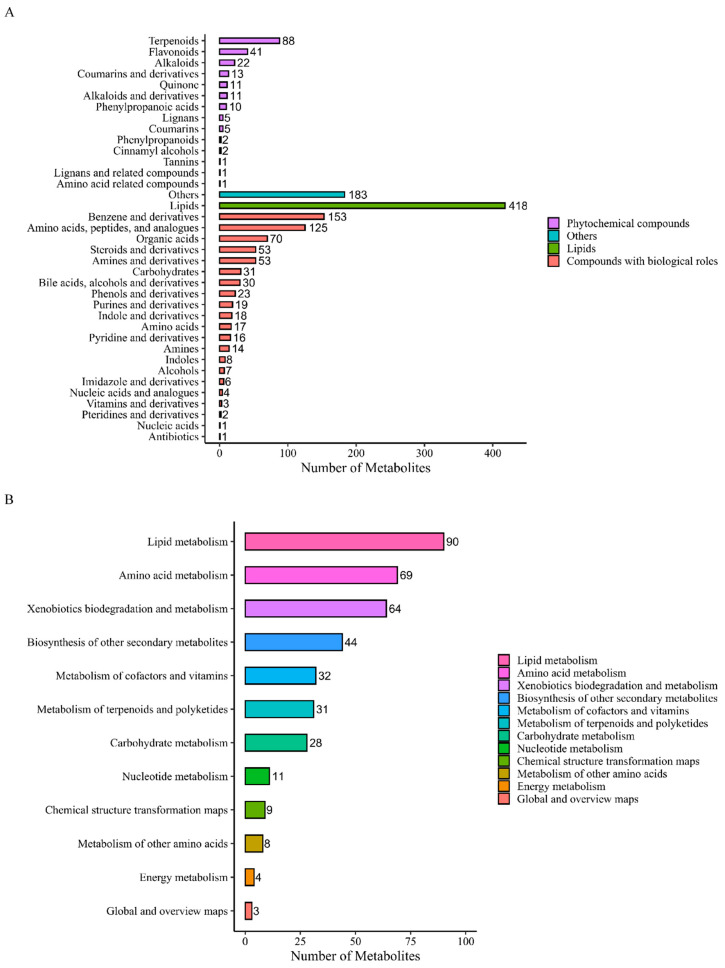

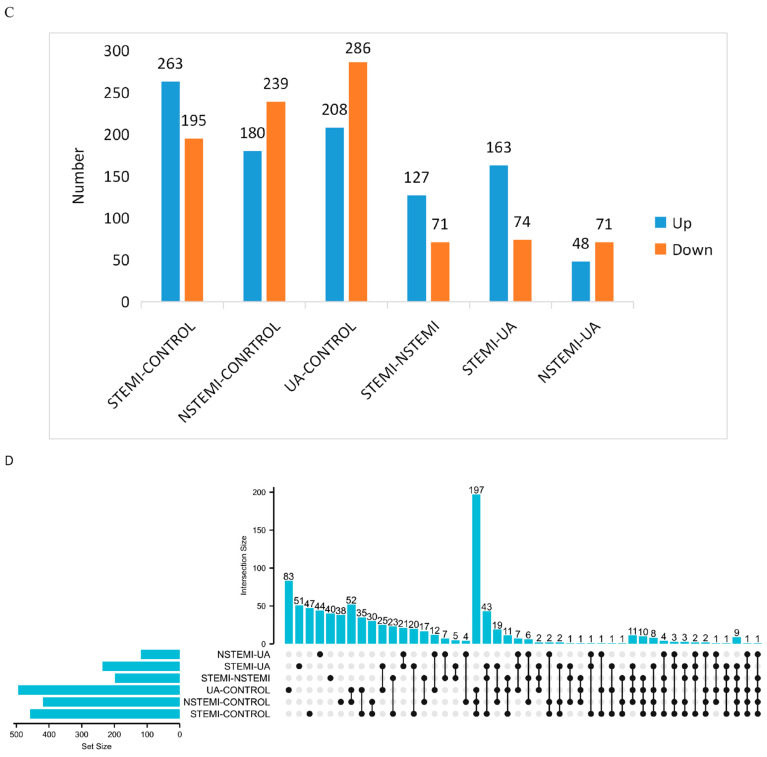

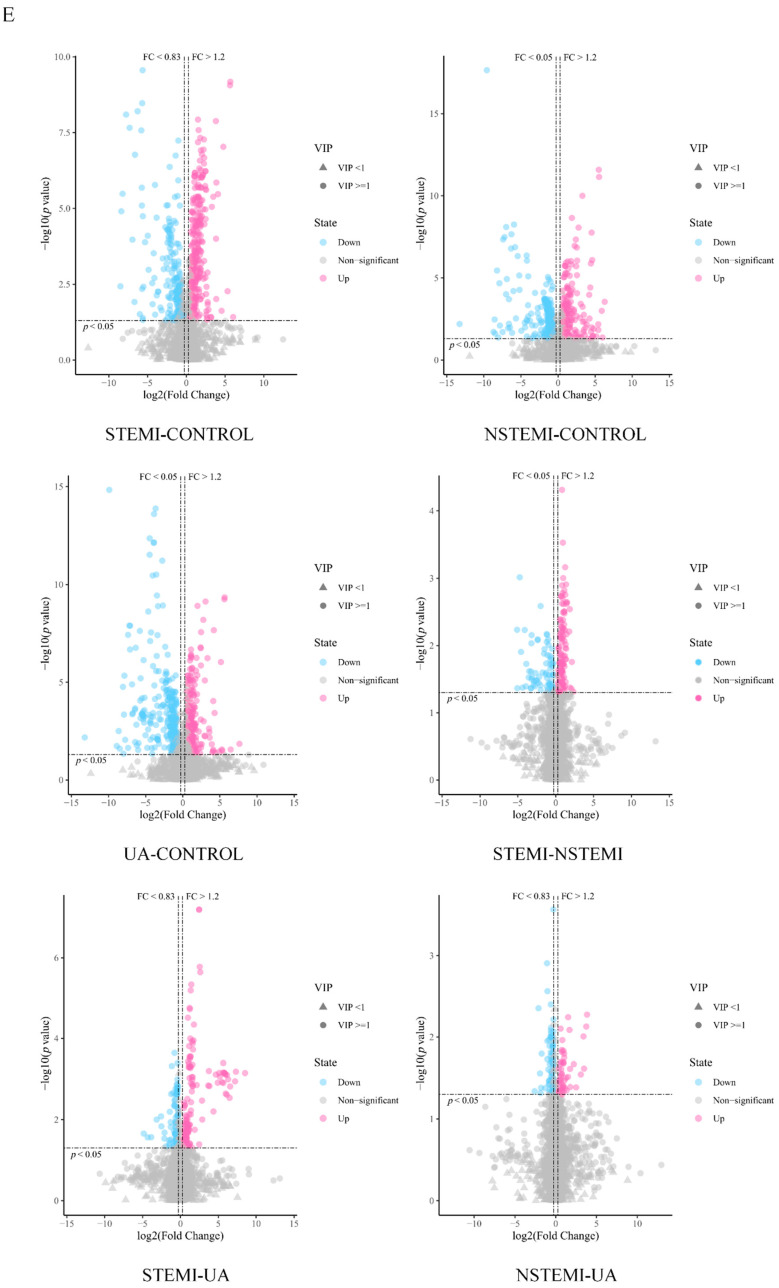

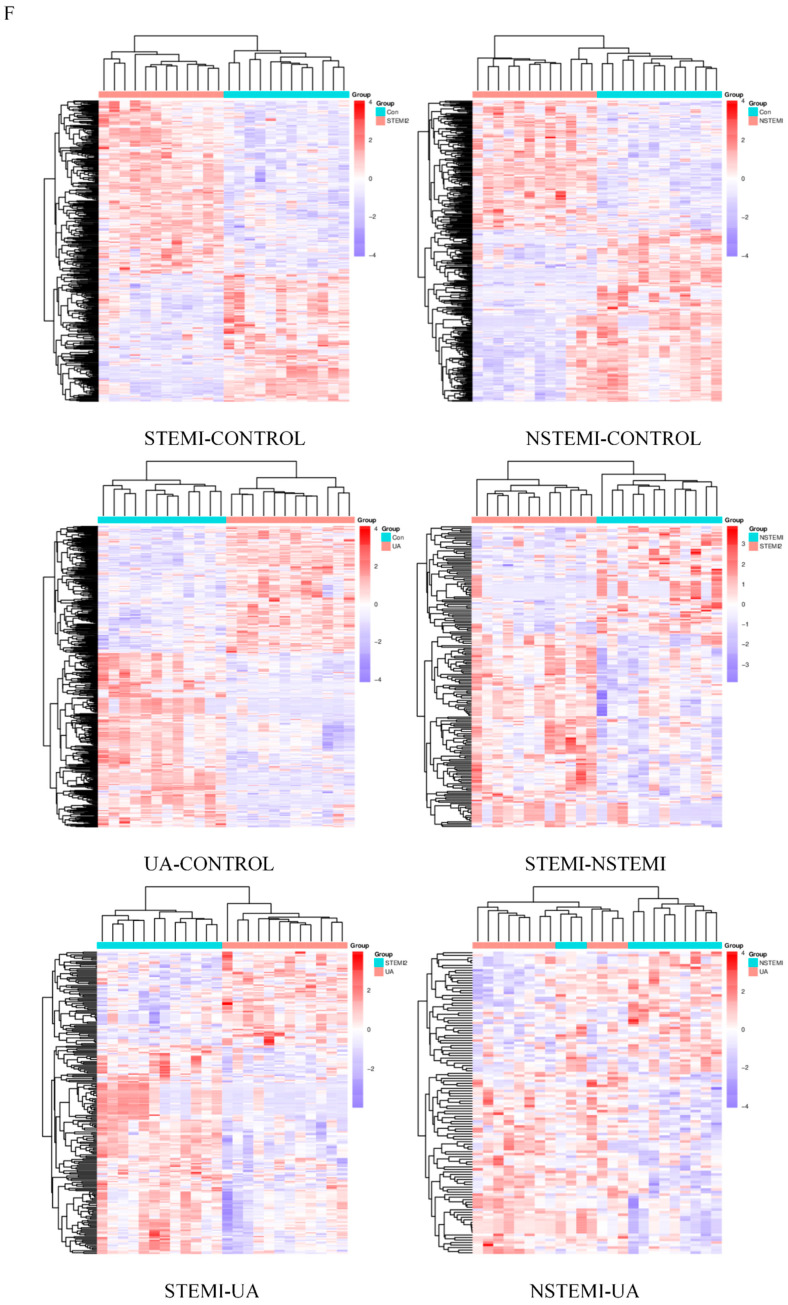

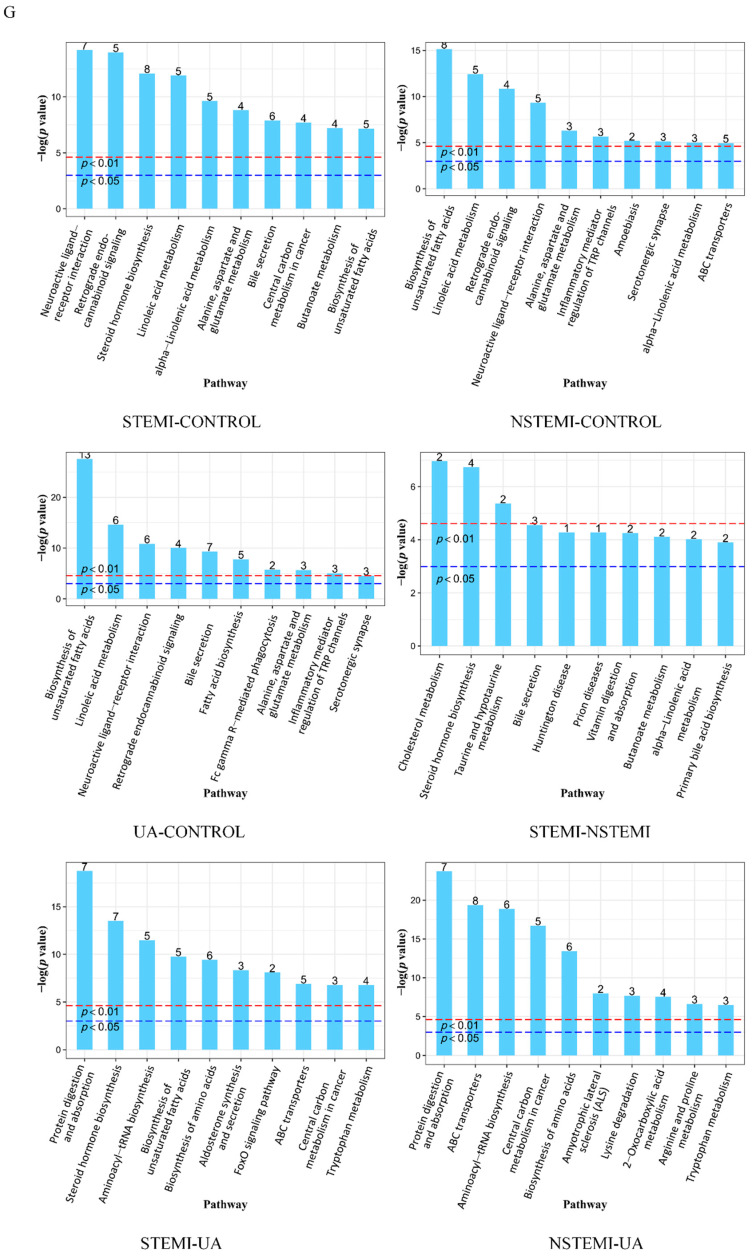

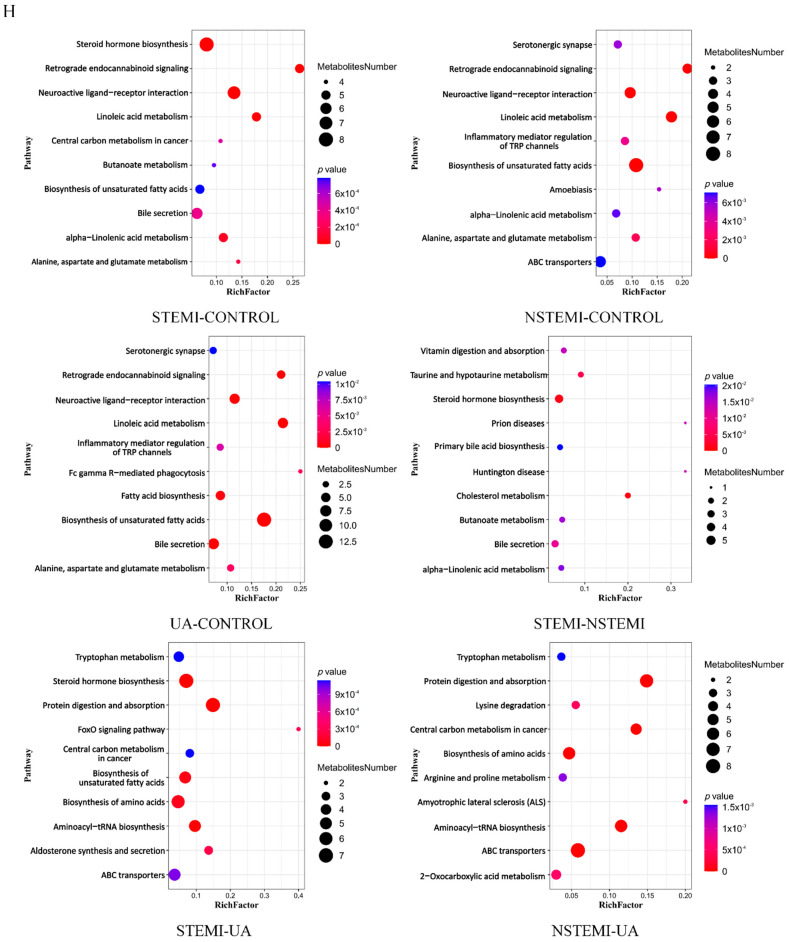
Cluster analysis and enrichment analysis of differential metabolites (DMs) in non-targeted metabolomics. (**A**) Metabolites classification bar chart. (**B**) Metabolic pathway classification bar chart. (**C**) Quantitative statistical chart of DMs. (**D**) UpSet diagram of DMs. (**E**) Volcanic map of DMs. (**F**) Cluster analysis heatmap of DMs. (**G**) Column diagram of metabolic pathway enrichment analysis. (The top 10 metabolic pathways with the smallest *p* value were drawn as column charts) (**H**) Bubble diagram of metabolic pathway enrichment analysis. (The X-axis Rich Factor is the number of DMs annotated in this pathway divided by all identified metabolites annotated in this pathway. The higher the value is, the higher the ratio of the DMs annotated in this pathway. The dot size represents the number of DMs annotated in this pathway).

In the discovery phase (STEMI, NSTEMI, UA, CTRL), a total of 2531 metabolites including 1925 DMs were identified, according to FC, *p* value, and VIP. These metabolites can be roughly divided into the following four categories: compounds with biological roles, lipids, phytochemical compounds, and others (Figure 2A), and were enriched in the 12 pathways of KEGG (Figure 2B). Figure 2C,D show the specific results. Figure 2E is a volcano map based on the DMs between each of the paired groups.

#### 3.2.2. Cluster Analysis and Correlation Analysis of DMs

Figure 2F is a cluster analysis heatmap of the DM expression levels in each of the comparisons. Supplementary Figure S1 provides the results of correlation analysis of the DMs.

#### 3.2.3. Metabolic Pathway Enrichment Analysis of DMs

Figure 2G,H analyze the metabolic pathways of DMs based on the KEGG database. Figure 2G shows the top ten metabolic pathways with the smallest *p* value in each comparison, and the number of the same DMs enriched in each pathway. Figure 2H shows the proportion, number, and *p* value of the metabolites annotated to different pathways. Supplementary Table S2 summarizes the top three metabolic pathways and the largest proportion and number of enriched DM pathways in each comparison.

### 3.3. Target Metabolomics Analysis Revealed the Potential Biomarkers of STEMI and NSTEMI Patients in the Validation Phase

**Figure 3:**
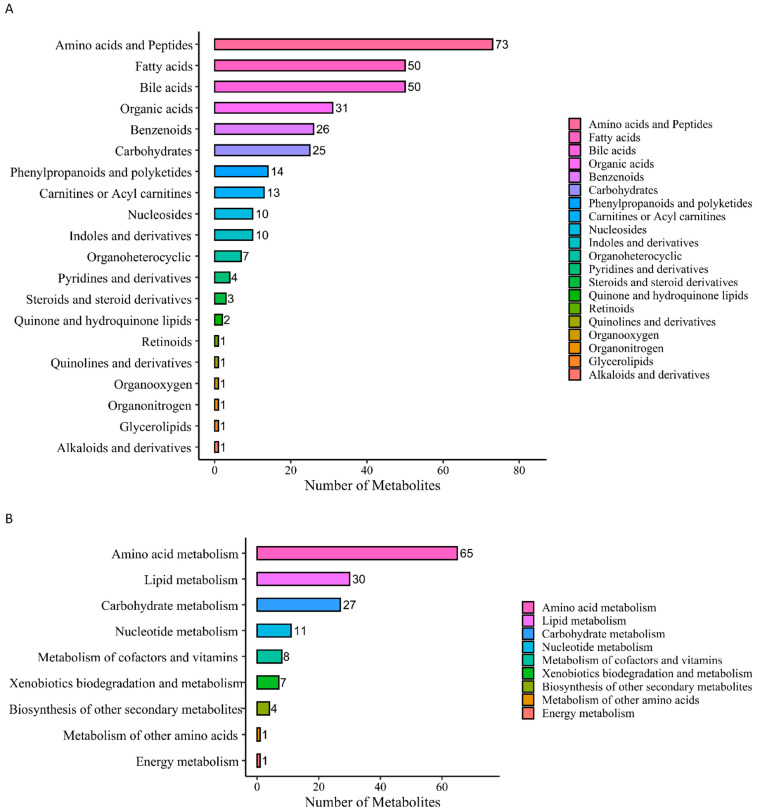

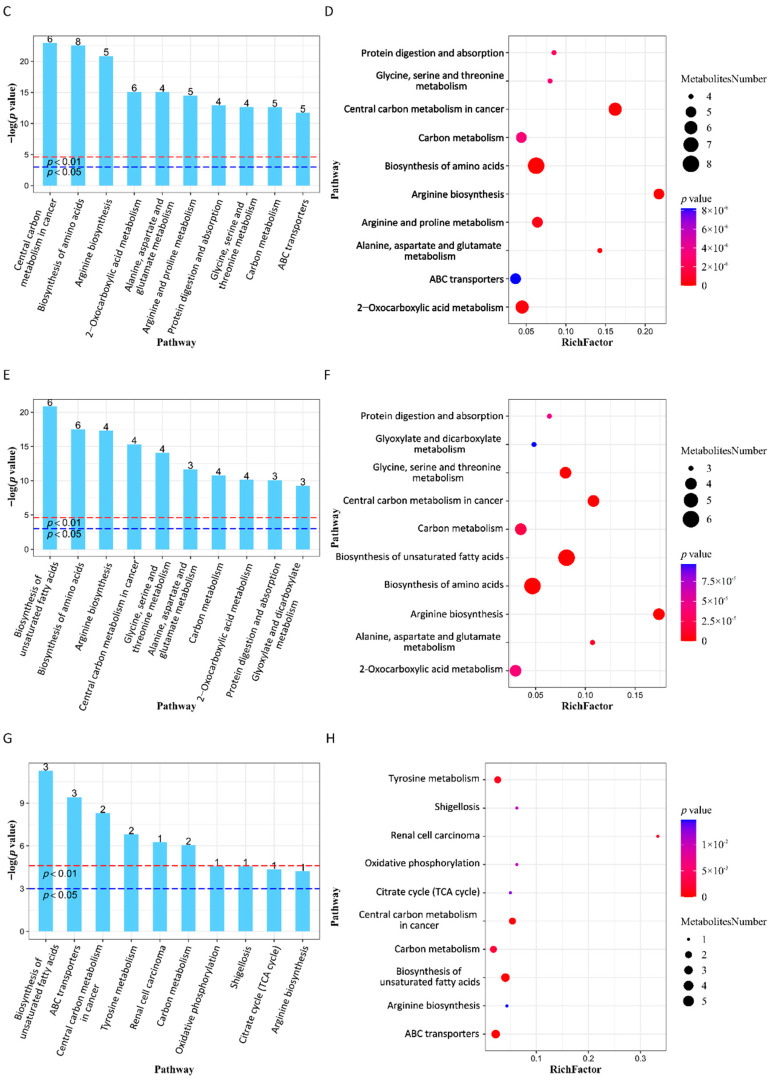
Cluster and enrichment analysis of differential metabolites verified by targeted metabolomics. (**A**) Metabolite classification bar chart. (**B**) Metabolic pathway classification bar chart. (**C**) Column chart of metabolic pathway enrichment analysis of “STEMI–CTRL”. (**D**) Bubble diagram of metabolic pathway enrichment analysis of “STEMI–CTRL”. (**E**) Column chart of metabolic pathway enrichment analysis of “NSTEMI–CTRL”. (**F**) Bubble diagram of metabolic pathway enrichment analysis of “NSTEMI–CTRL”. (**G**) Column chart of metabolic pathway enrichment analysis of “STEMI–NSTEMI”. (**H**) Bubble diagram of metabolic pathway enrichment analysis of “STEMI–NSTEMI”.

There were 27 DMs selected from the discovery phase for further validation using a HM Meta 700 panel (BGI, Shenzhen, China) that including 700 metabolites. In this validation phase, 324 metabolites were identified. These metabolites were divided into 20 categories (Figure 3A), among which amino acids and peptides, fatty acids, bile acids, organic acids, benzenoids, and carbohydrates were the main categories and included 255 metabolites, accounting for 78.7% of the total metabolites. In the KEGG analysis, a total of 154 metabolites were enriched in nine metabolic pathways (Figure 3B).

Among 324 metabolites, 61 DMs (57 up, 4 down) between the CTRL and STEMI groups, 41 DMs (28 up, 13 down) between then CTRL and NSTEMI groups, 20 DMs (17 up, 3 down) between the STEMI and NSTEMI groups were identified, respectively.

Figure 3C–H analyze metabolic pathways of DMs based on the KEGG database. Figure 3C,E,G show the top ten metabolic pathways with the smallest *p* value in each control group, and the number of the same DMs concentrated in each pathway. Figure 3D,F,H show the proportions, quantities, and *p* value of metabolites annotated to different pathways. Supplementary Table S3 summarizes the top three metabolic pathways and the largest proportion and number of enriched DM pathways in each comparison.

**Figure 4:**
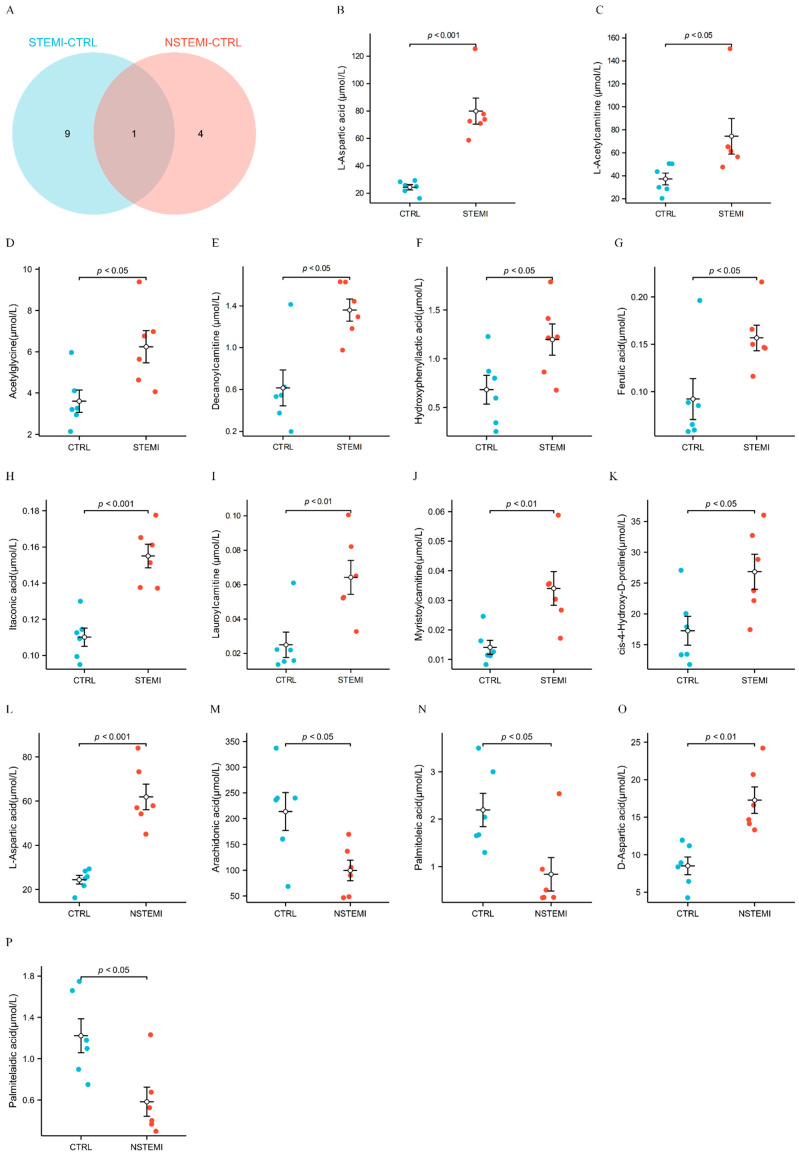

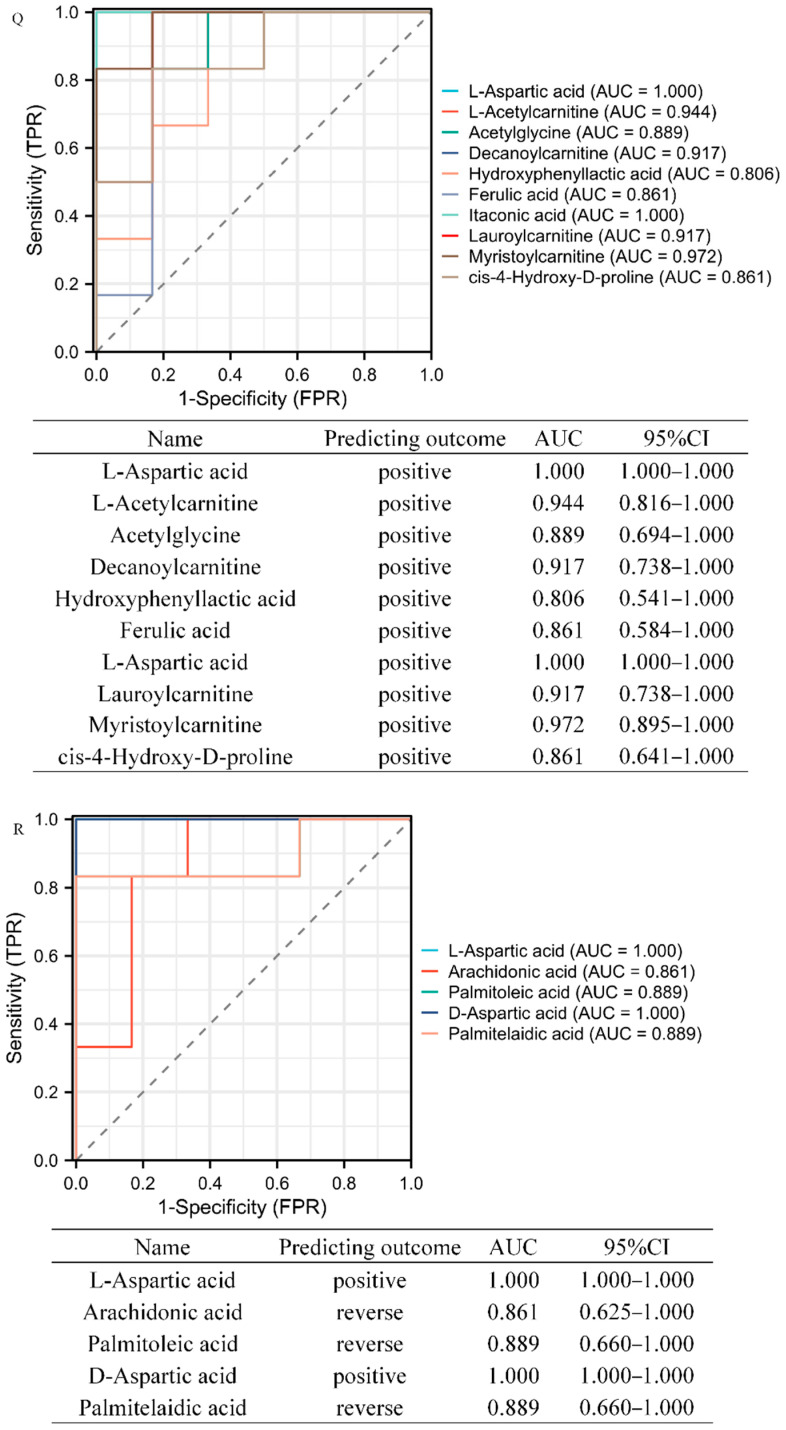
Wayne diagram, grouping comparison diagram and ROC analysis of the 15 differential metabolites (DMs). (**A**) Wayne diagram of DMs. (**B**–**K**) Grouping comparison chart of DMs expression in “STEMI–CTRL” group. (**L**–**P**) Grouping comparison chart of DMs expression in “NSTEMI–CTRL” group. (For (**B**–**P**), the error lines represent the mean ± standard error (SEM), respectively.) (**Q**) ROC curve chart of “STEMI–CTRL”. (**R**) ROC curve chart of “NSTEMI–CTRL”. (For (**Q**,**R**), “positive” stands for DMs’ upward adjustment and “reverse” stands for DMs’ downward adjustment).

Among these DMs, 14 were successfully verified by matching the DMs found in the discovery phase (Table 2, Figure 4A). Supplementary Figure S2 describes the mass spectrum diagram. 

Figure 4B–K are grouping comparison diagrams of 10 DMs in the “STEMI–CTRL” comparison, showing that the expression level in the STEMI group is significantly higher than in the CTRL group. Similarly, Figure 4L–P compare five DMs in the “NSTEMI–CTRL” comparison, showing that the expression levels of L-aspartic acid and D-aspartic acid in the NSTEMI group are significantly higher than in the CTRL group. Further, the expression levels of arachidonic acid, palmitoleic acid, and palmitelaidic acid in the NSTEMI group are significantly lower than those in the CTRL group.

Figure 4Q,R are ROC graphs drawn by grouping these 14 DMs. All AUC values are more than 0.8.

### 3.4. The Correlation between the DMs and CKMB

The expressions of L-aspartic acid, L-acetylcarnitine, and myristoylcarnitine in the STEMI group were significantly positively correlated with CKMB value, while other DMs were not significantly correlated with the CKMB value. The linear regression analysis data of the DMs and CKMB are shown in the Supplementary Table S4.

### 3.5. The Correlation between the DMs and the Severity of MI

In this study, the vessel lesions of the samples were counted, and the “single-vessel disease” was classified as “not severe”, while the “multi-vessel disease” was classified as “severe”. Through the regression analysis of DM expression and disease severity in the validation cohort samples, the results showed that there was no significant correlation between DMs and disease severity (*p* > 0.05). The linear regression analysis data of the DM expression and severity are shown in the Supplementary Table S5.

### 3.6. The Correlation between the DMs and Risk Factors

By using the regression analysis of DM expression and risk factors in the patients, the results show that there is no significant correlation between DM expression and risk factors, except hydroxyphenylacetic acid. The linear regression analysis data of the DMs and risk factors are shown in the Supplementary Table S6.

## 4. Discussion

**Figure 5:**
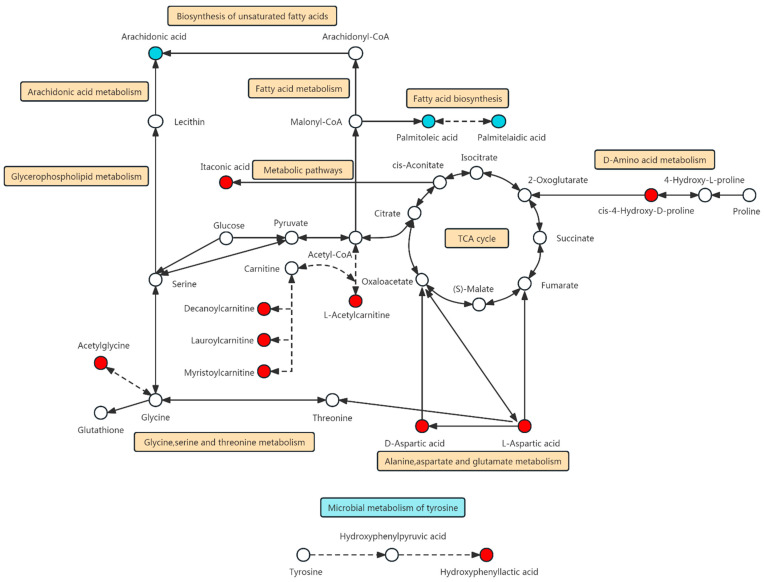
Diagram of the integrated metabolic pathways of the 14 differential metabolites in myocardial infarction discovered from this study. The metabolic pathway integration map contains 11 metabolic pathways of 13 differential metabolites (metabolic pathway of ferulic acid related biological system was not found in the database), including the following 10 modified KEGG maps: “hsa00470 D-Amino acid metabolism”, “hsa00020 Citrate cycle (TCA cycle)”, “hsa00061 Fatty acid biosynthesis”, “hsa00250 Alanine, aspartate, and glutamate metabolism”, “hsa00260 Glycine, serine, and threonine metabolism”, “hsa01100 Metabolic pathways”, “hsa01040 Biosynthesis of unsaturated fatty acids”, “hsa01212 Fatty acid metabolism”, “hsa00564 Glycerophospholipid metabolism”, “hsa00590 Arachidonic acid metabolism” and 1 pathway obtained from the literature review, “Microbial metabolism of tyrosine. (Dots represent metabolites, red is upregulated and blue is downregulated. The line represents the metabolic pathway, the straight line is quoted by KEGG, and the dotted line is quoted by literature review).

The present study using metabolomics analysis has found the following: (1) there were 14 DMs in ACS patients during the first 2–8 h from the onset of the chest pain; (2) there were 10 DMs for STEMI and 5 DMs for NSTEMI with high sensitivity and specificity; (3) these DMs may be developed as biomarker for the early diagnosis of acute MI; and (4) the DMs are included in several metabolic pathways (Figure 5) that may reveal the mechanism of the production and effect of the DMs found in this study.

### 4.1. L-Acetylcarnitine, Decanoylcarnitine (C10), Lauroylcarnitine (C12), and Myristoylcarnitine (C14)

Fatty acids are the main oxidative fuel of the heart, contributing 70–80% of the energy related to mitochondrial oxidative phosphorylation and ATP production [20]. L-carnitine (carnitine) is an endogenous cofactor, which can mediate the entry of fatty acids into the mitochondria, promote the β-oxidation of fatty acids in mitochondria, and achieve the balance of cardiac energy metabolism [21].

**Figure 6:**
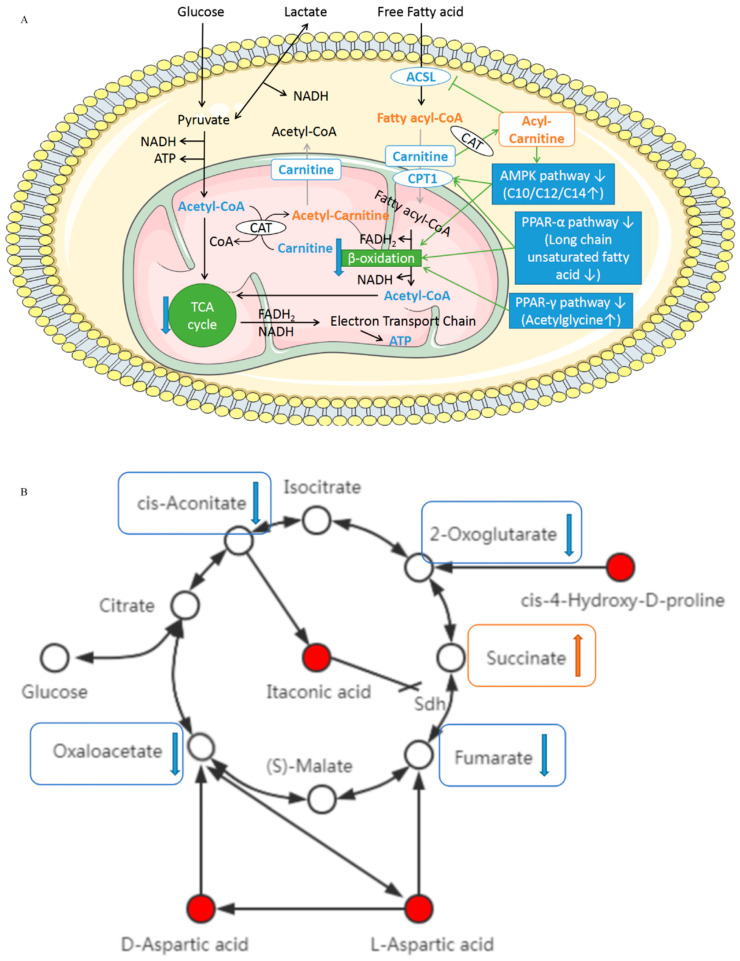

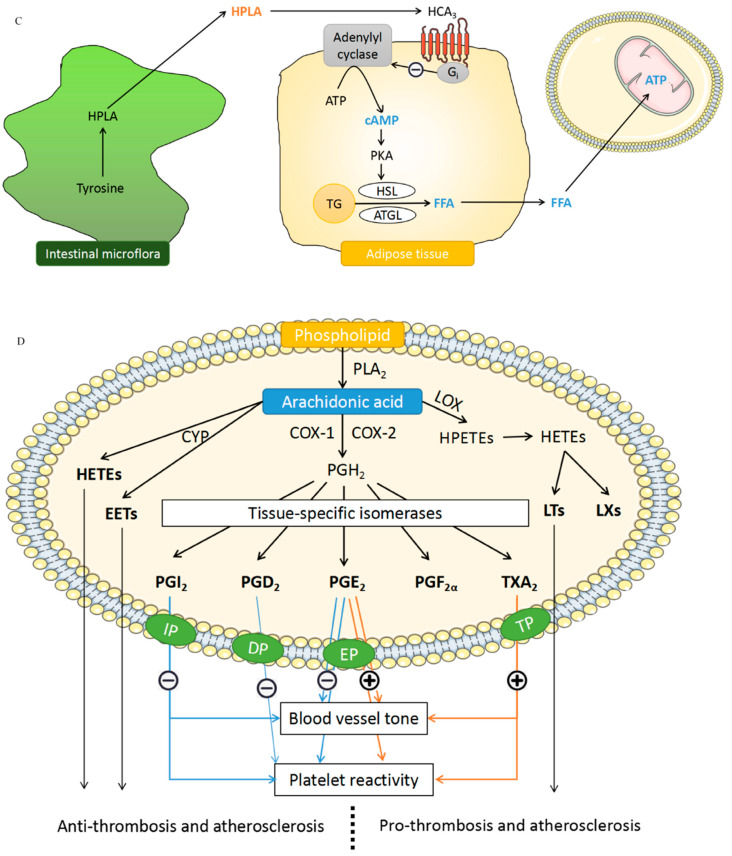
Schematic diagram of mechanism of action for the differential metabolites (DMs). (**A**) Schematic diagram of the influence mechanism of the DMs on intracellular fatty acid metabolism, β-oxidation, and TCA cycle. (C10: decanoylcarnitine; C12: lauroylcarnitine; C14: myristoylcarnitine; ACSL: long-chain isomer of acetyl-CoA synthetase; CPT1: carnitine palmitoyl transferase 1; CAT: carnitine acyltransferase.) (**B**) Direct influence of DMs on TCA cycle and schematic diagram of related mechanisms. (Sdh: succinate dehydrogenase.) (**C**) Schematic diagram of the mechanism of action of hydroxyphenylacetic acid. (HPLA: hydroxyphenyllactic acid; FFA: free fatty acids). (**D**) Schematic diagram of the mechanism of action of arachidonic acid. (COX: cyclooxygenase, CYP: cytochrome P450 enzyme, LOX: lipid oxygenase, HETEs: hydroxyeicosatetraenoic acid, LTs: leukotriene, LXs: lipoxin, EETs: epoxeicosatrienoic acid.) [Some of the pictures in this figure are from smart.servier.com] (accessed on 6 July 2023).

During hypoxia and ischemia, the production of acylcarnitine causes the consumption of free carnitine in the cells [21]. As shown in Figure 6A, the shuttle of mitochondrial carnitine is interrupted and the fatty acid metabolism, β-oxidation activity, and acetyl-CoA production decrease. Thus, the activity of TCA cycle reduces and eventually leads to a decrease in ATP production, destruction of cardiac energy metabolism balance, and low myocardial function.

Secondly, the accumulation of medium- and long-chain acylcarnitine also damages the long-chain isomer of acetyl-CoA synthetase (ACSL) that is responsible for catalyzing the activation of fatty acids [22]. The decrease in ACSL affects fatty acid biosynthesis and metabolism, with adverse effects on energy production and cell survival.

In addition, the accumulation of medium- and long-chain acylcarnitine also causes the disorder of adenosine 5′-monophosphate-activated protein kinase (AMPK) signaling pathway, leading to the increase in lipid deposition in myocardial cells, and eventually causing myocardial lipotoxicity, hypertrophy, fibrotic remodeling, and increased apoptosis [23,24,25]. 

Finally, the incomplete β-oxidation due to the accumulation of acylcarnitine and the decrease in acetyl-CoA, as a result of the decrease in TCA cycle activity, destroys the balance of cardiac energy metabolism.

### 4.2. L-aspartic Acid, D-aspartate, cis-4-hydroxy-D-proline, and Itaconic Acid

Figure 6B shows the metabolic relationship between L-aspartic acid, D-aspartate, cis-4-hydroxy-D-proline, itaconic acid, and the TCA cycle. The increase in these four DMs directly leads to the decrease in related substances in the TCA cycle. Moreover, the accumulation of itaconic acid inhibits the activity of succinate dehydrogenase (Sdh) [26]. The above results affect or even interrupt the TCA cycle and ultimately affect the energy metabolism of the heart.

### 4.3. Long Chain Monounsaturated Fatty Acids (Palmitoleic Acid/Palmitelaidic Acid)

Peroxisome proliferator-activated receptor (PPAR) is expressed in tissues with high fatty acid oxidation capacity, in which PPARα plays an important role in regulating the uptake, activation, and β-oxidation of fatty acids by the cells [16,27,28]. The natural preferential binding ligands of PPARα are long-chain unsaturated fatty acids [28,29], such as palmitoleic acid (16:1N-7). When the content of palmitoleic acid decreases, the induction of the PPARα signaling pathway is hindered, which weakens fatty acid oxidation and affects energy supply.

### 4.4. Acetylglycine

Glycine is necessary for collagen synthesis, and the defect of collagen in the arterial wall accelerates the calcification of the arterial middle layer [30]. Moreover, glycine can increase β-oxidation activity by activating the PPARγ signaling pathway and has anti-atherosclerosis and anti-inflammatory properties [31]. The increased acetylglycine found in this study may lead to a decrease in glycine, which typically inhibits PPARγ. It has been reported that the inhibition of PPARγ indicates that there is inflammation and oxidative stress [32].

### 4.5. Hydroxyphenyllactic Acid (HPLA)

HPLA is produced by the human intestinal flora through tyrosine metabolism [33]. HCA_3_ is a G_i_ protein-coupled receptor that plays an important role in maintaining energy homeostasis [34]. As shown in Figure 6C, HPLA activates the HCA_3_ receptor, inhibits adenylate cyclase, and reduces cAMP level [35]. The decrease in cAMP leads to low free fatty acids (FFA) and thus reduces the production of ATP. In addition, HCA_3_ also leads to the negative feedback mechanism of β-oxidation of FFA [34].

### 4.6. Arachidonic Acid (AA)

AA regulates complex cardiovascular functions under physiological and pathological conditions. As shown in Figure 6D, AA can be metabolized by cyclooxygenase (COX), cytochrome P450 enzyme (CYP), and lipid oxygenase (LOX). The various AA metabolites produced regulate different signal pathways by binding to different receptors [36].

### 4.7. Study Limitations

This study identified, for the first time, a number of metabolites in MI patients (including STEMI and NSTEMI). However, for clinical use, a large clinical trial should be arranged to verify the potential use of these DMs for the early diagnosis of STEMI and NSTEMI.

## 5. Conclusions

In this metabolomics study, we identified 14 DMs that may be developed as biomarkers for the early diagnosis of MI with high sensitivity and specificity at the early onset time. In particular, L-aspartic acid, arachidonic acid, palmitoleic acid, D-aspartic acid, and palmitelaidic acid as potential biomarkers for NSTEMI may have important clinical significance in NSTEMI patients because they have no typical ECG changes and these biomarkers may help in identifying these patients for adequate early management. Therefore, the present study has provided new information on the diagnosis of MI with important clinical significance.

## Figures and Tables

**Table 1:** Baseline characteristics for selected patients.

	Control	STEMI	NSTEMI	UA	*p*
	(*n* = 18)	(*n* = 18)	(*n* = 18)	(*n* = 12)	
Age, median (IQR)	64.00 (55.75–69.25)	67.00 (58.00–70.50)	66.50 (60.00–72.00)	63.50 (54.75–69.25)	0.728
Male, *n* (%)	9 (50%)	9 (50%)	9 (50%)	6 (50%)	1.000
BMI, kg/m^2^, median (IQR)	24.49 (23.43–25.55)	25.24 (21.17–28.04)	25.71 (22.63–27.89)	26.30 (23.89–29.84)	0.312
**Risk factors, *n* (%)**					
Hypertension, *n* (%)	11 (61.1%)	12 (66.7%)	11 (61.1%)	6 (50.0%)	0.839
Diabetes, *n* (%)	1 (5.6%)	7 (38.9%)	9 (50.0%)	5 (41.7%)	0.020
Dyslipidemia, *n* (%)	2 (11.1%)	8 (44.4%)	5 (27.8%)	5 (41.7%)	0.112
Current smoker, *n* (%)	4 (22.2%)	6 (33.3%)	4 (22.2%)	3 (25.0%)	0.878
**Cardiac Biomarkers, median (IQR)**					
Myo (ng/mL)	NA	333.00 (190.00–500.00)	213.50 (123.25–408.50)	74.20 (62.60–86.55)	<0.001
CKMB (ng/mL)	NA	4.50 (2.85–26.22)	6.25 (2.50–8.55)	1.25 (1.00–1.68)	0.001
hs-cTnI (ng/mL)	NA	0.145 (0.055–1.308)	0.425 (0.180–0.958)	0.050 (0.050–0.050)	0.002
BNP (pg/mL)	NA	30.15 (21.00–48.58)	57.75 (44.62–221.75)	32.40 (8.50–95.00)	0.062
D-Dimer (ng/mL)	NA	124.5 (100.0–409.2)	164.5 (100.0–545.5)	134.5 (100.0–224.0)	0.608
**Vessel lesion, *n* (%)**					
Single-vessel disease	0 (0%)	1 (5.6%)	8 (44.4%)	2 (16.7%)	0.020
Multi-vessel disease	0 (0%)	17 (94.4%)	10 (55.6%)	10 (83.3%)	0.020
**Laboratory data**					
Blood glucose (mmol/L)	5.25 (5.10–5.68)	8.05 (6.70–10.18)	7.35 (6.32–9.48)	7.25 (5.88–7.92)	<0.001
ALT (U/L)	16.50 (13.25–30.25)	34.50 (29.00–80.25)	17.50 (13.00–24.50)	16.50 (10.00–20.00)	<0.001
AST (U/L)	17.00 (16.00–23.25)	114.00 (72.50–349.00)	31.00 (24.50–58.00)	18.00 (14.50–19.50)	<0.001
AST/ALT	1.10 (0.80–1.20)	3.45 (3.05–4.60)	2.15 (1.30–3.60)	1.05 (0.90–1.35)	<0.001
GGT (U/L)	22.50 (13.25–51.50)	20.50 (16.00–30.50)	21.00 (17.00–31.00)	25.50 (20.7–32.50)	0.824
TP (g/L)	68.50 (67.00–71.50)	64.00 (60.00–67.75)	64.00 (62.25–67.25)	63.00 (62.00–64.25)	0.022
TBIL (μmol/L)	9.15 (7.02–14.48)	12.40 (10.52–20.72)	9.55 (8.42–12.15)	10.10 (6.05–13.42)	0.094
ALP (U/L)	78.50 (66.00–87.50)	72.00 (66.00–91.00)	70.00 (62.50–95.25)	66.50 (63.25–81.75)	0.741
Urea (mmol/L)	6.00 (4.90–7.05)	5.55 (4.98–6.32)	7.00 (5.70–7.70)	5.80 (4.90–7.30)	0.138
Uric acid (μmol/L)	299.50 (255.00–326.25)	323.00 (249.25–355.75)	285.00 (246.25–379.75)	296.00 (252.00–381.50)	0.982
CREA (μmol/L)	57.50 (50.75–65.75)	61.00 (48.00–76.00)	58.00 (50.25–94.25)	63.00 (60.25–67.25)	0.866
Total cholesterol (mmol/L)	4.70 (4.08–5.00)	4.90 (4.18–5.65)	4.30 (3.50–5.10)	4.15 (3.80–4.80)	0.080
TG (mmol/L)	1.16 (0.94–1.40)	1.28 (1.16–1.87)	1.61 (1.06–2.44)	1.91 (1.54–2.18)	0.032
HDL-C (mmol/L)	1.18 (0.99–1.39)	1.11 (1.01–1.17)	0.92 (0.70–1.08)	0.88 (0.80–0.94)	0.001
LDL-C (mmol/L)	2.98 (2.60–3.25)	3.10 (2.72–3.80)	2.86 (2.06–3.52)	2.47 (2.21–3.10)	0.111
PLT (×10^9^/L)	223.00 (209.00–272.50)	244.00 (206.25–270.00)	224.50 (198.00–250.25)	243.00 (203.00–261.50)	0.733
**Length of stay (days)**	1.00 (1.00–2.00)	8.00 (6.25–9.75)	7.00 (6.00–8.00)	3.00 (2.75–6.50)	<0.001

Data are expressed as median (IQR) or numbers (percentage). Abbreviation: BMI, body mass index; Myo, myoglobin; CKMB, creatine kinase myocardial band; hs-cTnI, high-sensitivity cardiac troponin I; BNP, B-type natriuretic peptide; ALT, alanine aminotransferase; AST, aspartate aminotransferase; GGT, gamma-glutamyl transferase; TP, total protein; TBIL, total bilirubin; ALP, alkaline phosphatase; CREA, creatinine; TG, triglyceride; HDL-C, high-density lipoprotein cholesterol; LDL-C, low-density lipoprotein cholesterol; PLT, platelet count.

**Table 2:** The specific information of 14 differential metabolites that were successfully verified.

Group	HMDB ID	Name	Discovery Group			Validation Group		
			FC	*p* Value	VIP	FC	*p* Value	VIP
CONTROL-STEMI	HMDB0000191	L-Aspartic acid	2.11358712	8.21 × 10^−7^	1.577611655	3.279994663	7.22 × 10^−6^	1.896413607
	HMDB0000201	L-Acetylcarnitine	1.965571929	5.54 × 10^−5^	1.340201316	1.997169295	0.013503462	1.253073946
	HMDB0000532	Acetylglycine	1.725948017	0.002633113	1.245356672	1.733124121	0.013522338	1.431596275
	HMDB0000651	Decanoylcarnitine	4.27951669	0.000127514	1.844505879	2.213434982	0.013596305	1.628532799
	HMDB0000755	Hydroxyphenyllactic acid	2.714464103	1.94 × 10^−6^	1.813677974	1.752930832	0.049981208	1.281225837
	HMDB0000954	Ferulic acid	6.08440531	0.024210222	1.290022785	1.699855439	0.019853603	1.375369857
	HMDB0002092	Itaconic acid	1.598398693	2.34 × 10^−5^	1.168632741	1.407598002	0.000256761	1.869606847
	HMDB0002250	Lauroylcarnitine	2.749056744	0.000898345	1.466835554	2.565912117	0.004524507	1.522012879
	HMDB0005066	Myristoylcarnitine	2.166262401	2.10 × 10^−6^	1.516728633	2.413711584	0.002971447	1.533423213
	HMDB0060460	cis-4-Hydroxy-D-proline	1.86876251	4.64 × 10^−6^	1.416576047	1.553709509	0.023058005	1.359347074
CONTROL-NSTEMI	HMDB0000191	L-Aspartic acid	1.989941591	4.99 × 10^−6^	1.411097512	2.540095524	2.64 × 10^−5^	2.282586177
	HMDB0001043	Arachidonic acid	0.409790387	0.000367677	1.480786535	0.465412831	0.034645824	1.677437241
	HMDB0003229	Palmitoleic acid	0.407686414	0.002118188	1.451292166	0.382412824	0.01157985	1.670575527
	HMDB0006483	D-Aspartic acid	1.532946834	6.03 × 10^−5^	1.053107988	2.024616281	0.003515567	2.032273973
	HMDB0012328	Palmitelaidic acid	0.557037574	0.001707426	1.109067827	0.476871871	0.01046257	1.749465707

## Data Availability

The data are available from the corresponding author upon reasonable request.

## Supplementary Materials

The following supporting information can be downloaded at: https://www.mdpi.com/article/10.3390/biom14050532/s1, Figure S1: Correlation chord diagrams on differential metabolites; Figure S2: Mass spectra of 14 differential metabolites; Table S1: Multiple hypothesis test of factors with significant differences; Table S2: Summary of DMs pathways of discovery phase; Table S3: Summary of DMs pathways of validation phase; Table S4: Regression analysis of DMs and CKMB; Table S5: Regression analysis of DMs and disease severity; Table S6: Regression analysis of DMs and risk factors.
