# Supplementary material for: Metabolomics Analysis Identifies Differential Metabolites as Biomarkers for Acute Myocardial Infarction
Source: Biomolecules. 2024 Apr 29;14(5):532. doi: 10.3390/biom14050532 (PMC11117998; doi:10.3390/biom14050532)
Supplement: Supplementary file 1 [file biomolecules-14-00532-s001.zip › biomolecules-2893417-supplementary.pdf]

Figure S1 Correlation chord diagrams on differential metabolites.

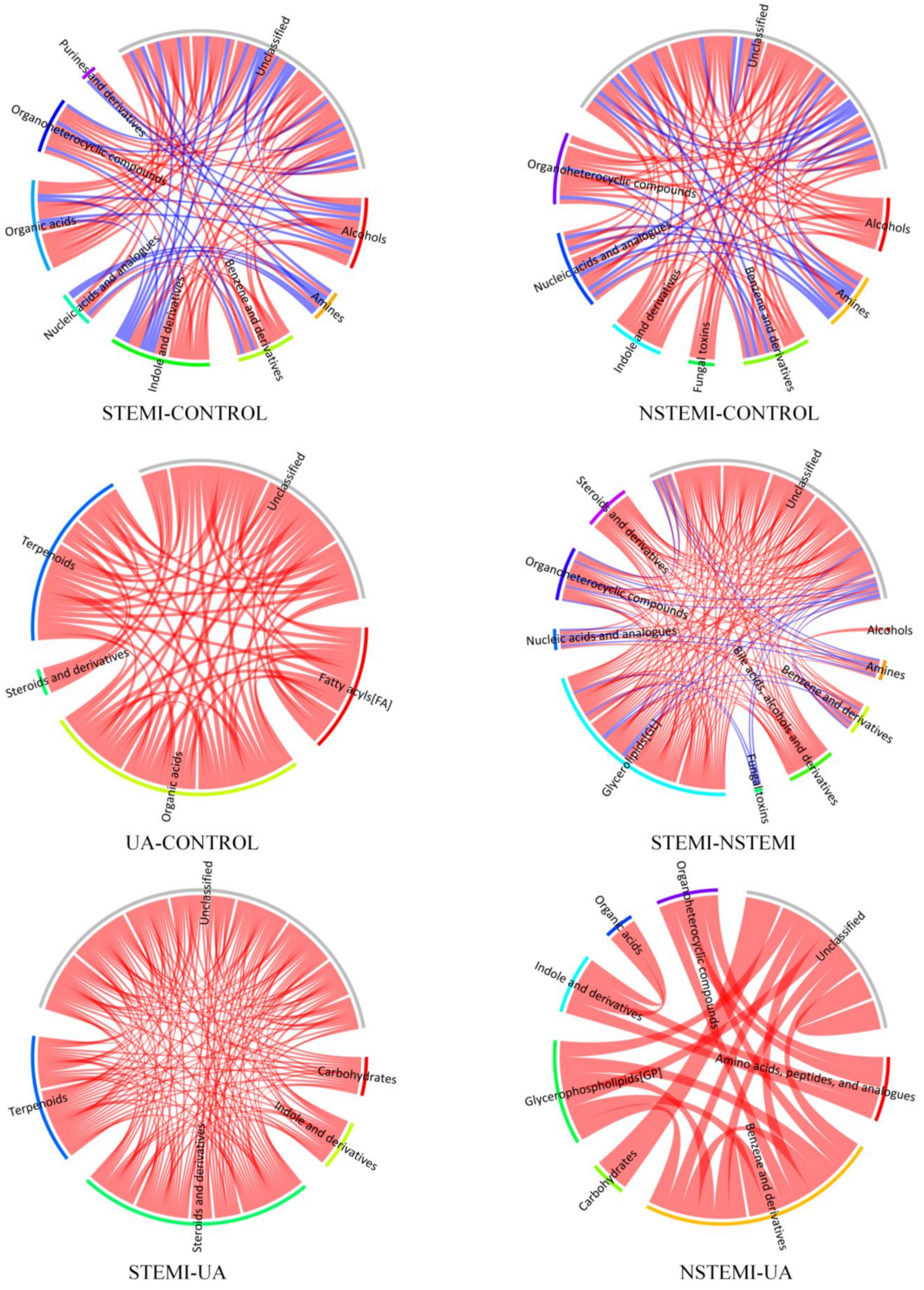

Figure S2 Mass spectra of 14 differential metabolites.

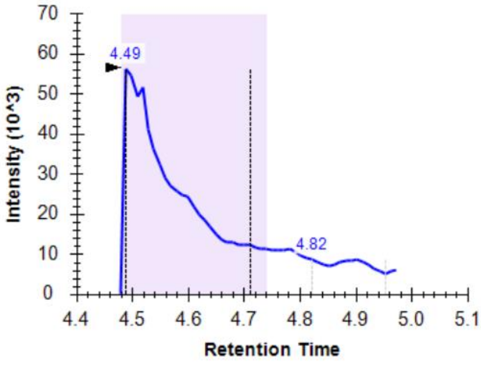

L-Aspartic acid

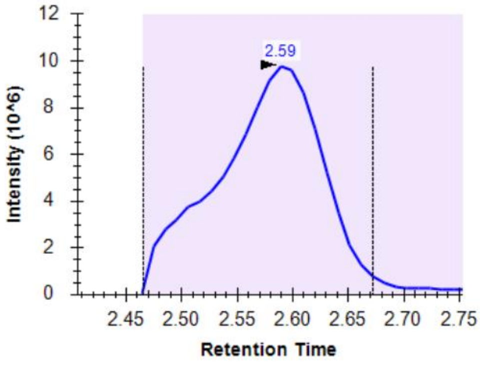

L-Acetylcarnitine

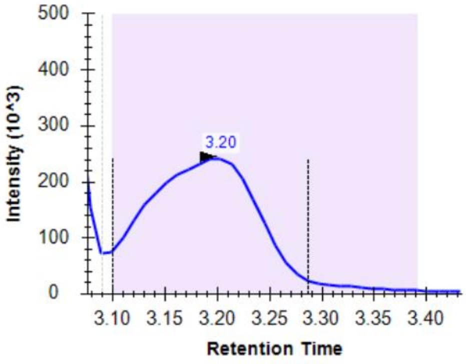

Acetylglycine

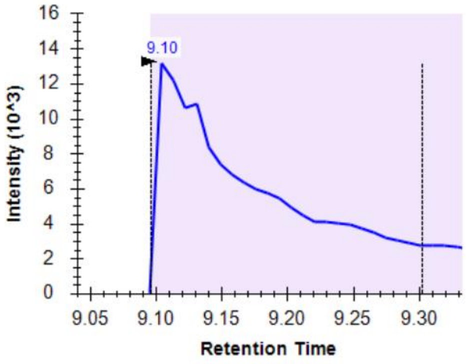

Decanoylcarnitine (C10)

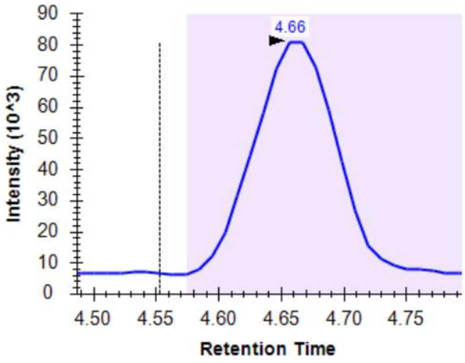

Hydroxyphenyllactic acid

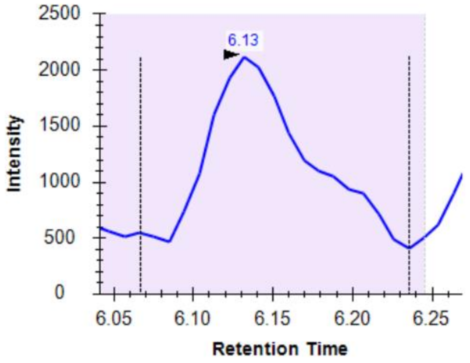

Ferulic acid

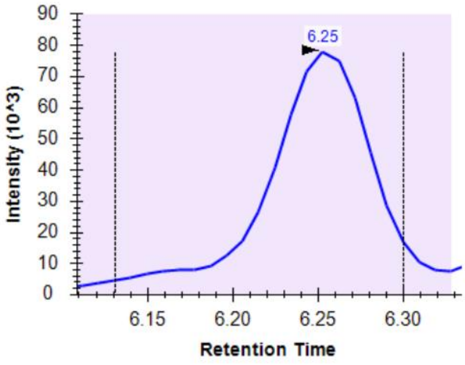

Itaconic acid

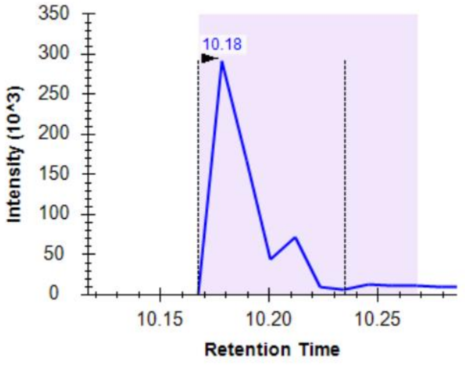

Lauroylcarnitine

Figure S2 Continued

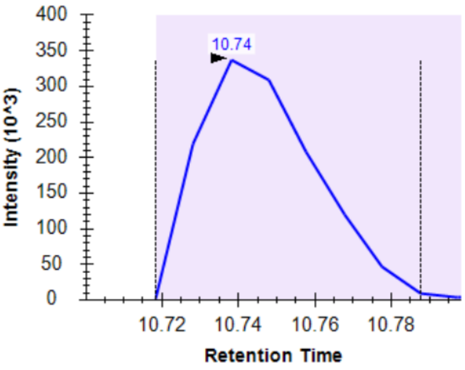

Myristoylcarnitine

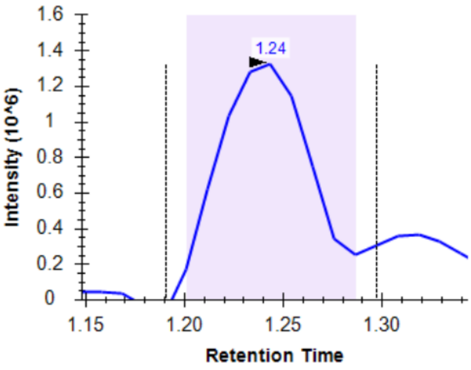

cis-4-Hydroxy-D-proline

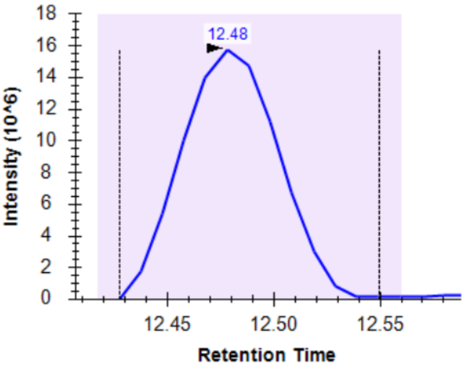

Arachidonic acid

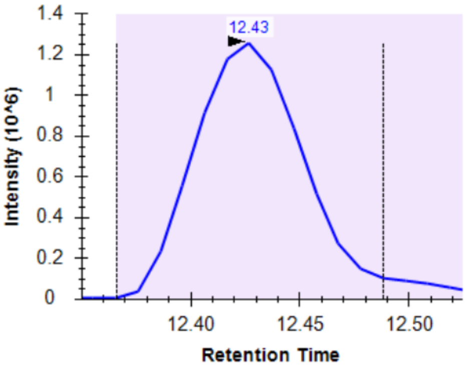

Palmitoleic acid

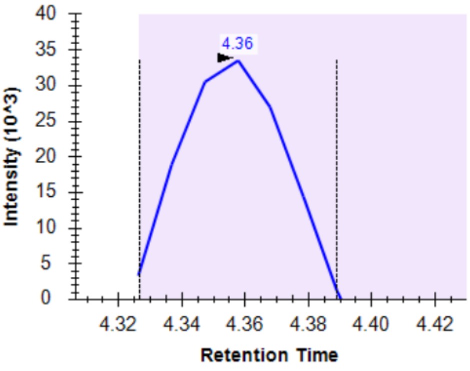

D-Aspartic acid

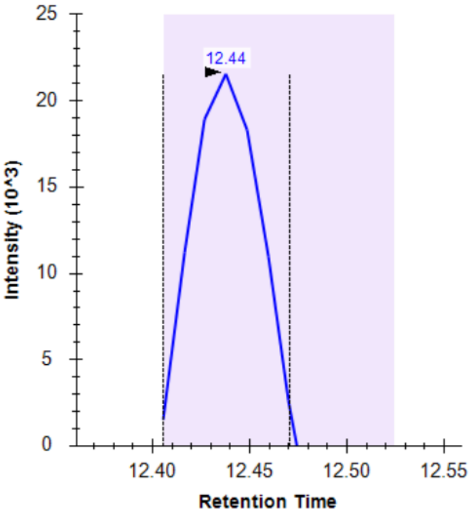

Palmitelaidic acid

Table S1 Multiple hypothesis test of factors with significant differences.

	group I	group J	p-adjusted
**Cardiac Biomarkers**			
Myo	STEMI	NSTEMI	0.579
	STEMI	UA	<0.001
	NSTEMI	UA	0.006
CKMB	STEMI	NSTEMI	1.000
	STEMI	UA	0.002
	NSTEMI	UA	0.005
TnI	STEMI	NSTEMI	1.000
	STEMI	UA	0.017
	NSTEMI	UA	0.002
**Laboratory data**			
Blood glucose	control	STEMI	<0.001
	control	NSTEMI	<0.001
	control	UA	0.014
	STEMI	NSTEMI	1.000
	STEMI	UA	0.972
	NSTEMI	UA	1.000
ALT	control	STEMI	0.017
	control	NSTEMI	1.000
	control	UA	1.000
	STEMI	NSTEMI	0.004
	STEMI	UA	0.001
	NSTEMI	UA	1.000
AST	control	STEMI	<0.001
	control	NSTEMI	0.017
	control	UA	1.000
	STEMI	NSTEMI	0.06
	STEMI	UA	<0.001
	NSTEMI	UA	0.014
ALT/AST	control	STEMI	<0.001
	control	NSTEMI	<0.001
	control	UA	1.000
	STEMI	NSTEMI	0.681
	STEMI	UA	<0.001
	NSTEMI	UA	0.035
TP	control	STEMI	0.166
	control	NSTEMI	0.175
	control	UA	0.028
	STEMI	NSTEMI	1.000

	STEMI	UA	1.000
	NSTEMI	UA	1.000
TG	control	STEMI	0.538
	control	NSTEMI	0.216
	control	UA	0.030
	STEMI	NSTEMI	1.000
	STEMI	UA	1.000
	NSTEMI	UA	1.000
HDL-C	control	STEMI	0.665
	control	NSTEMI	0.008
	control	UA	0.006
	STEMI	NSTEMI	0.137
	STEMI	UA	0.084
	NSTEMI	UA	0.967
**Length of stay**			
	control	STEMI	<0.001
	control	NSTEMI	<0.001
	control	UA	0.101
	STEMI	NSTEMI	1.000
	STEMI	UA	0.032
	NSTEMI	UA	0.169

Table S2 Summary of DMs pathways of discovery phase.

Group	TOP3 pathway	DMs number	TOP1 DMs proportion pathway	TOP1 DMs number pathway
STEMI-CTRL	Neuroactive ligand-receptor interaction（hsa04080）	7	Retrograde endocannabinoid signaling（hsa04723）	Steroid hormone biosynthesis（hsa00140）
	Retrograde endocannabinoid signaling（hsa04723）	5		
	Steroid hormone biosynthesis（hsa00140）	8		
NSTEMI-CTRL	Biosynthesis of unsaturated fatty acids（hsa01040）	8	Retrograde endocannabinoid signaling（hsa04723）	Biosynthesis of unsaturated fatty acids（hsa01040）
	Linoleic acid metabolism（hsa00591）	5		
	Retrograde endocannabinoid signaling（hsa04723）	4		
UA-CTRL	Biosynthesis of unsaturated fatty acids（hsa01040）	13	Fc gamma R-mediated phagocytosis（hsa04666）	Biosynthesis of unsaturated fatty acids（hsa01040）
	Linoleic acid metabolism（hsa00591）	6		
	Neuroactive ligand-receptor interaction（hsa04080）	6		
STEMI-NSTEMI	Cholesterol metabolism（hsa04979）	2	Prion diseases（hsa05020）<br>Huntington disease（hsa05016）	Steroid hormone biosynthesis（hsa00140）
	Steroid hormone biosynthesis（hsa00140）	4		
	Taurine and hypotaurine metabolism（hsa00430）	2		
STEMI-UA	Protein digestion and absorption（hsa04974）	7	FoxO signaling pathway（hsa04068）	Protein digestion and absorption（hsa04974）<br>Steroid hormone biosynthesis（hsa00140）
	Steroid hormone biosynthesis（hsa00140）	7		
	Aminoacyl-tRNA biosynthesis（hsa00970）	5		
NSTEMI-UA	Protein digestion and absorption（hsa04974）	7	Amyotrophic lateral sclerosis (ALS)（hsa05014）	ABC transporters（hsa02010）
	ABC transporters（hsa02010）	8		
	Aminoacyl-tRNA biosynthesis（hsa00970）	6		

Table S3 Summary of DMs pathways of validation phase.

Group	TOP3 pathway	DMs number	TOP1 DMs proportion pathway	TOP1 DMs number pathway
STEMI-CTRL	Central carbon metabolism in cancer（hsa05230）	6	Arginine biosynthesis（hsa00220）	Biosynthesis of amino acids（hsa01230）
	Biosynthesis of amino acids（hsa01230）	8		
	Arginine biosynthesis（hsa00220）	5		
NSTEMI-CTRL	Biosynthesis of unsaturated fatty acids（hsa01040）	6	Arginine biosynthesis（hsa00220）	Biosynthesis of unsaturated fatty acids（hsa01040）<br>Biosynthesis of amino acids（hsa01230）
	Biosynthesis of amino acids（hsa01230）	6		
	Arginine biosynthesis（hsa00220）	4		
STEMI-NSTEMI	Biosynthesis of unsaturated fatty acids（hsa01040）	3	Renal cell carcinoma（hsa05211）	Biosynthesis of unsaturated fatty acids（hsa01040）<br>ABC transporters（hsa02010）
	ABC transporters（hsa02010）	3		
	Central carbon metabolism in cancer（hsa05230）	2		

Table S4 Regression analysis of DMs and CKMB.

		CKMB	
		P	Coefficients
STEMI	L-Aspartic acid	0.002	1.893
	L-Acetylcarnitine	0.001	3.121
	Acetylglycine	1.000	-
	Decanoylcarnitine	0.287	-
	Hydroxyphenyllactic acid	0.295	-
	Ferulic acid	0.793	-
	Itaconic acid	0.175	-
	Lauroylcarnitine	0.082	-
	Myristoylcarnitine	0.013	0.001
	cis-4-Hydroxy-D-proline	0.188	-
NSTEMI	L-Aspartic acid	0.291	-
	Arachidonic acid	0.391	-
	Palmitoleic acid	0.526	-
	D-Aspartic acid	0.438	-
	Palmitelaidic acid	0.494	-

Table S5 Regression analysis of DMs and disease severity.

		P value severity
STEMI	L-Aspartic acid	All the samples were multi-vessel diseases, and there was no difference in severity.
	L-Acetylcarnitine	
	Acetylglycine	
	Decanoylcarnitine	
	Hydroxyphenyllactic acid	
	Ferulic acid	
	Itaconic acid	
	Lauroylcarnitine	
	Myristoylcarnitine	
	cis-4-Hydroxy-D-proline	
NSTEMI	L-Aspartic acid	0.397
	Arachidonic acid	0.893
	Palmitoleic acid	0.473
	D-Aspartic acid	0.266
	Palmitelaidic acid	0.391

Table S6 Regression analysis of DMs and risk factors.

		P value			
		Hypertension	Diabetes	Dyslipidemia	Smoke
STEMI	L-Aspartic acid	0.382	0.476	0.334	0.438
	L-Acetylcarnitine	0.432	0.840	0.598	0.504
	Acetylglycine	0.801	0.970	0.824	0.760
	Decanoylcarnitine	0.665	0.431	0.984	0.740
	Hydroxyphenyllactic acid	0.002	0.002	0.002	0.007
	Ferulic acid	0.673	0.582	0.618	0.665
	Itaconic acid	0.198	0.597	0.558	0.648
	Lauroylcarnitine	0.468	0.552	0.773	0.965
	Myristoylcarnitine	0.373	0.848	0.570	0.909
NSTEMI	cis-4-Hydroxy-D-proline	0.271	0.273	0.095	0.115
	L-Aspartic acid	0.485	0.472	0.716	0.791
	Arachidonic acid	0.792	0.675	0.603	0.723
	Palmitoleic acid	0.998	0.674	0.839	0.171
	D-Aspartic acid	0.509	0.476	0.886	0.931
	Palmitelaidic acid	0.844	0.601	0.666	0.196
